# The prevalence, diagnostic accuracy and genotype-phenotype correlation of *GNAS* mutations in fibrous dysplasia: a meta-analysis

**DOI:** 10.3389/fgene.2024.1377716

**Published:** 2024-07-29

**Authors:** Ao-Bo Zhang, Jian-Yun Zhang, Jiang Xue, Zhen-Chao Wu, Zhi-Xiu Xu, Li-Sha Sun, Tie-Jun Li

**Affiliations:** ^1^ Department of Oral Pathology, Peking University School and Hospital of Stomatology and National Center of Stomatology and National Clinical Research Center for Oral Diseases & National Engineering Research Center of Oral Biomaterials and Digital Medical Devices, Beijing, China; ^2^ Research Unit of Precision Pathologic Diagnosis in Tumors of the Oral and Maxillofacial Regions, Chinese Academy of Medical Sciences (2019RU034), Beijing, China; ^3^ Department of Pulmonary and Critical Care Medicine, Peking University Third Hospital, Beijing, China; ^4^ Central Laboratory, Peking University School and Hospital of Stomatology, Beijing, China; ^5^ National Clinical Research Center for Oral Diseases and National Engineering Laboratory for Digital and Material Technology of Stomatology and Beijing Key Laboratory of Digital Stomatology, Beijing, China

**Keywords:** *GNAS*, fibrous dysplasia, diagnostic accuracy, meta-analyses, McCune Albright

## Abstract

**Background:**

There is inconsistent evidence regarding the accuracy of *GNAS* mutations identification for the diagnosis of FD/MAS. This study was performed to estimate the prevalence and diagnostic accuracy of *GNAS* mutations detection and to preliminarily investigate the genotype-phenotype correlation in FD patients.

**Methods:**

Five electronic databases were searched from 1995 to 2024 using search terms related to *GNAS* and fibrous dysplasia. Observational studies of FD patients undergoing *GNAS* mutation detection in FD were included.

**Results:**

A total of 878 FD patients were included. The pooled prevalence of *GNAS* mutations in FD based on the random effects model was 74% (95% CI = 64%–83%). Regarding diagnostic accuracy, a sensitivity of 0.83 (95% CI, 0.65–0.96), specificity of 0.99 (95% CI, 0.98–1.00) and the area under the receiver operating characteristic curve of 98.38% were found. Additionally, meta-analysis and Fisher’s test showed the *GNAS* mutation types were significantly associated with FD types (OR = 3.51, 95% CI = 1.05 to 11.72; *p* < 0.05).

**Conclusion:**

A high detection rate of *GNAS* mutations occurred in FD, and its detection is reliable for diagnosing FD. Additionally, *GNAS* mutation type was types were significantly associated with FD type.

**Systematic Review Registration:**

Identifier CRD42024553469.

## 1 Introduction

Fibrous dysplasia (FD) is a rare, genetic but noninheritable bone disorder caused by a postzygotic mutation of *GNAS* located on chromosome 20q13.3 (Weinstein et al., 1991), which presents in three forms: monostotic FD, which occurs in one bone; polyostotic FD, which involves multiple bones; and McCune–Albright syndrome, which simultaneously contains polyostotic FD, skin hyperpigmentation and hyperfunctioning endocrinopathies (Collins et al., 2012). In this disorder, nonfunctional fibrous tissue with an abnormal quality and structure replaces normal bone tissue, which can cause pain and bone deformities and increase the risk of fracture, and these manifestations initially occur during childhood (Liens et al., 1994).

The GNAS mutations identified in FD patients result in a significantly elevated level of cyclic adenosine monophosphate (cAMP) in FD patients. As a result, this mutation is regarded as the leading cause of FD (Parvanescu et al., 2014). The specific location of the mutation is arginine 201 in exon region 8, which is usually substituted by either histidine (R201H) or cysteine (R201C). However, it is unclear whether different *GNAS* mutation variants could influence the regulation of GαS activity to different extents. Zhadina et al. found no significant genotype-phenotype correlation for the severity of FD in a retrospective analysis of clinical data obtained as part of a long-standing natural history study (Zhadina et al., 2021). However, the relevant evidence has not been further documented by new studies.

In addition, several studies identified *GNAS* mutations within FD, but other diseases that need to be distinguished from FD, for example, ossifying fibroma (OF) and low-grade central osteosarcoma (lgc-OSA), do not show *GNAS* mutations, which indicates the possible role of *GNAS* mutation analysis in the differential diagnosis of FD. However, without a comprehensive analysis with more data, it is still not clear what the exact prevalence and diagnostic accuracy of *GNAS* mutations detection are in FD.

In this study, we performed a meta-analysis to evaluate the prevalence and diagnostic accuracy of *GNAS* mutations detection in FD. We also investigated whether the different *GNAS* mutation types could be linked with the different FD forms. We hypothesized that *GNAS* mutation detection could be a very reliable test for FD diagnosis and possesses a very high diagnostic accuracy, especially specificity, and that the R201H mutation type is likely a predominant pathogenic variant in FD. To our knowledge, this study is the largest to date on the subject and the first undertaken to investigate the prevalence, diagnostic accuracy and genotype-phenotype correlation of *GNAS* mutations in FD to date. This study allows us to understand the prevalence and diagnostic accuracy of GNAS mutations detection in FD, which could help doctors distinguish FD from other diseases requiring differential diagnosis and prioritize variants that cause a greater burden of disease when developing targeted therapy for FD.

## 2 Materials and methods

### 2.1 Literature sources and search strategy

This study followed the Meta-analysis of Observational Studies in Epidemiology (MOOSE) guidelines from the screening protocol to the pooled analysis (Stroup et al., 2000) and was registered in PROSPERO (CRD42024553469). The literature search was performed in the PubMed, Web of Science, Cochrane Library and CNKI (China National Knowledge Infrastructure) databases to identify full-text articles up to 1 Jan 2024. The key search items were as follows: fibrous dysplasia, *GNAS*. There was no language restriction. Furthermore, the citations of potentially relevant studies from the retrieved articles were reviewed. The details of the PubMed and Web of Science search strategies are listed in the Supplement-Appendix as examples. Three independent investigators (Authors 1–3) screened each abstract against the inclusion criteria; conflicts resulted in inclusion at this stage. Full-text records for relevant abstracts were retrieved, and each was independently reviewed by two investigators (Author 1, 2). Two investigators then used standardized forms to extract the study endpoints.

### 2.2 Inclusion and exclusion criteria

The inclusion criteria for the studies to be considered in this study were as follows: (1) articles related to *GNAS* mutations in fibrous dysplasia; (2) studies with studies with five samples or >5 samples; and (3) studies that involved human subjects. The rationale behind establishing a threshold for sample size in included studies stems from the potential for a low sample size to introduce significant bias in prevalence estimates. In this study, we opted for “studies with five samples or >5” over a more stringent criterion of “studies with 10 samples or >10” due to our recognition of FD as a rare disease, which poses challenges in acquiring an adequate number of cases.

The exclusion criteria were as follows: (1) case reports, review articles and meeting abstracts; (2) animal studies; (3) studies with fewer than 5 FD cases; and (4) studies that did not provide essential information concerning sequencing methods and tissue sources.

### 2.3 Quality assessment

Quality assessments of the cross-sectional studies were performed according to the Agency for Healthcare Research and Quality (AHRQ) modified scale for observational studies, which includes 11 items (Rockville, 2008). For the 11 items, two reviewers (Authors 1 and 2) selected on of three options, including Yes, no and unclear. “Yes” was for one point, while “No” and “Unclear” were zero points (Zhou et al., 2021). The total score obtained was for the quality of each study (0–3: low quality; 4–7: moderate quality; 8–11: high quality). Additionally, the risk of bias (ROB) of the included diagnostic studies was determined by the Quality Assessment of Diagnostic Accuracy Studies-2 (QUADAS-2) tool (Whiting et al., 2011).

### 2.4 Data collection

According to a previously created data collection form, two reviewers (Author 1 and 2) independently extracted the data from the included studies. The following data were collected: the country of the study; authors and year of publication; the sample size of FD patients; incidence of *GNAS* positive mutation; FD types; FD lesion location; *GNAS* mutation detection methods; the tissue source of DNA extraction; and *GNAS* mutation types.

### 2.5 Statistical methods

Data analysis was performed with R version 4.2.0 and Review Manager 5.4.0. The “meta” (version 6.0, univariate model) and “metadiag4” (version 2.1.1, bivariate model) packages were used to calculate the pooled prevalence and diagnostic accuracy of *GNAS* mutations, respectively (Leeflang, 2014), and the Review Manager was used to analyze the genotype-phenotype correlation with odds ratios. The data on the prevalence of *GNAS* mutations were tested for normality. If the test showed a *p*-value > 0.05, the data were considered to conform to a normal distribution, and there was no need to convert the original prevalence data. However, if the test showed a *p*-value < 0.05, it was necessary to convert the data using methods such as logarithmic conversion, logit conversion, arcsine conversion and the Freeman-Tukey dual arcsine conversion methods; then, the normality of each conversion was verified in sequence until the converted data followed a standard normal distribution. Statistical heterogeneity was assessed by the *I* ^2^ test at *a* = 0.1. A random-effects model was adopted. The causes of heterogeneity were explored by meta-regression. We performed a subgroup analyses according to sample size, detection methods and source of DNA acquisition. The associations between genotypes and phenotypes were evaluated by Fisher’s exact test and meta-analysis. The statistical significance of the hypothesis test was set at *p* < 0.05, and 95% CIs were calculated using the Clopper-Pearson method. Forest plots were used to show pooled outcomes, and a summary receiver operating characteristic curve (SROC) was generated using a Bayesian bivariate hierarchical model.

## 3 Results

### 3.1 Characteristics of the included studies

**FIGURE 1:**
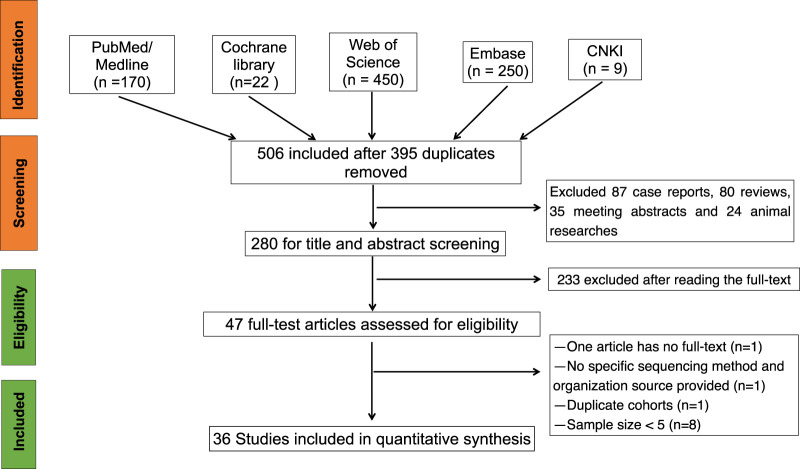
Flow diagram of searching process.

**FIGURE 2:**
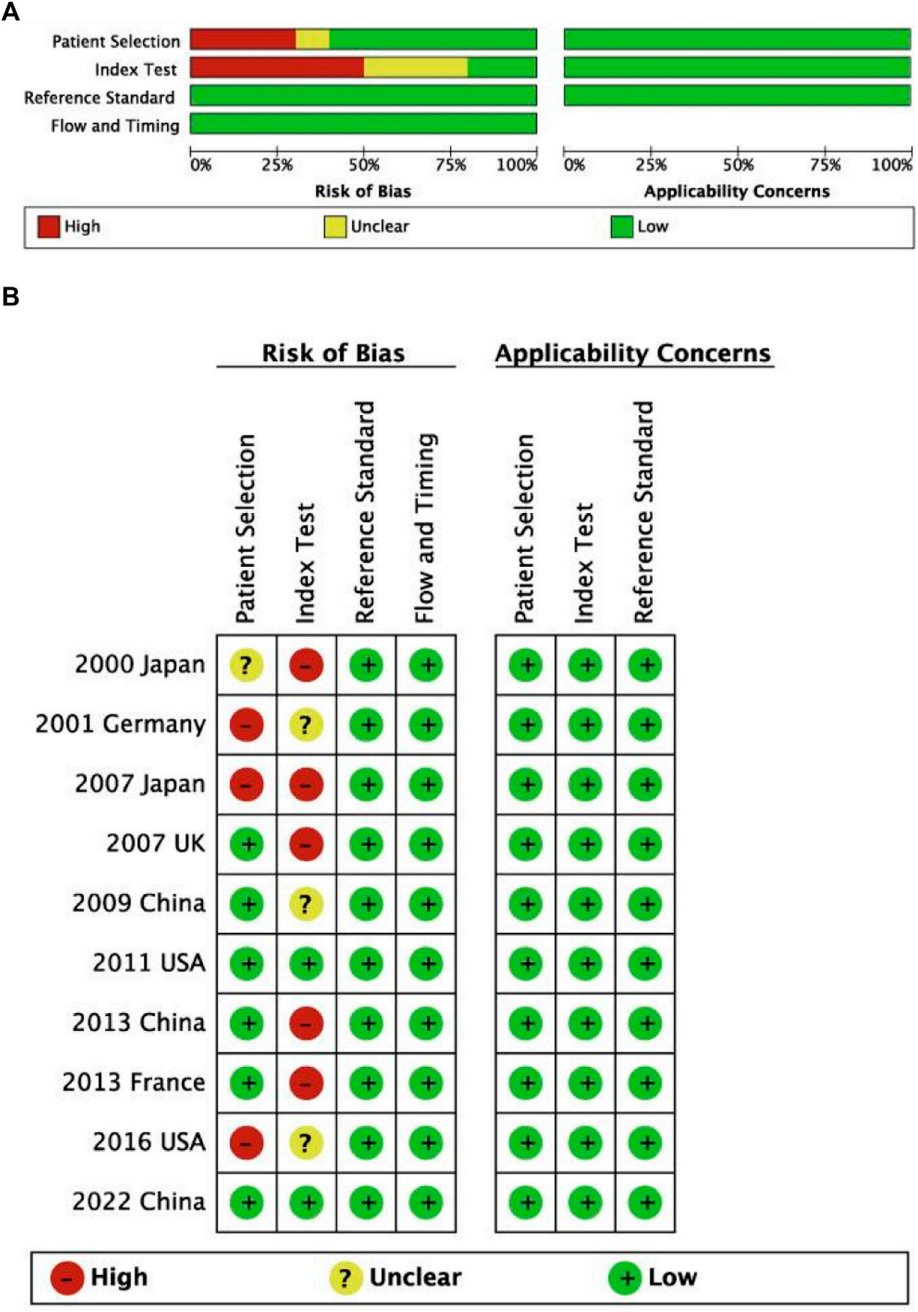
Risk of bias and applicability concerns for diagnostic studies on summary **(A)** and each included study **(B)** plots.

The literature search flow chart is shown in Figure 1. A total of 901 studies were obtained from five electronic databases (PubMed, 170; Cochrane, 22; Web of Science, 450; Embase, 250; CNKI, 9), of which 395 were duplicates. Then, 470 articles were excluded because they did not meet the inclusion criteria (Figure 1). Finally, 36 studies containing 878 FD patients were included in this meta-analysis after reading the full texts (Candeliere et al., 1997; Stanton et al., 1999; Bianco et al., 2000; Sakamoto et al., 2000; Pollandt et al., 2001; Corsi et al., 2003; Kalfa et al., 2006; Kobayashi et al., 2006; Idowu et al., 2007; Michienzi et al., 2007; Toyosawa et al., 2007; Kuznetsov et al., 2008; Juan et al., 2009; Liang et al., 2011; Mariot et al., 2011; Lee et al., 2012; Narumi et al., 2013; Shi et al., 2013; Tabareau-Delalande et al., 2013; Gaujoux et al., 2014; Walther et al., 2014; Cho et al., 2016; Jour et al., 2016; Xue-yan et al., 2016; Shin et al., 2017; Zuo and Zhang, 2017; Majoor et al., 2018; Author Anynomous, 2019; Elli et al., 2019; Majoor et al., 2019; Romanet et al., 2019; Hagelstein-Rotman et al., 2021; Zhadina et al., 2021; Hagelstein-Rotman et al., 2022; Xue et al., 2022; Roszko et al., 2023). Twenty-three studies employed conventional sequencing methods (CS), 21 of which of studies utilized direct sequencing and 2 of which studies employed clone sequencing. The remaining studies adopted modified sequencing approaches (MS), including next-generation sequencing (NGS, six studies), pyrophosphorolysis activated polymerization (PAP, three studies), and advanced PCR technology (four studies). In terms of DNA acquisition sources, 28 studies extracted DNA from bone tissue, six studies obtained DNA from blood, and two studies retrieved DNA from myxoma in patients with FD. All included articles were published from 1997 to 2023 and in English, except for three articles in Chinese. The characteristics of each study are presented in Table 1, and the AHRQ and QUADAS-2 quality assessments of the included studies are shown in the last column in Table 1 and Figure 2, respectively.

**TABLE 1:** Characteristics of the included studies.

Year/Author	Country (Publication)	Detection method	Tissue	Location (no.)	Total FD case (no.)	GNAS Mutation/FD cases (No.)	Genotype (No.)	Control group (No.)	FD type	AHRQ score
2023/Roszko	United States (Roszko etal., 2023)	ddPCR/castPCR	Blood	NA	66	45/66	R201H (32) R201C (12) R101C (1)	13 volunteer healthy samples	MFD:8 PFD:57	10/HQ
2022/Xue	China (Xue et al., 2022)	DS	Bone	CGN (26); CGN + EGN (3)	29	24/29	R201H (14) R201C (10)	DSOM (36)	MAS (3), MFD (14), PFD(12)	NA
2022/Rotman	France (Hagelstein-Rotman et al., 2022)	NGS	Bone	CGN + EGN (5) EGN (12)	17	13/17	R201H (6) R201C(7)	NA	MAS (7) MFD (2) PFD(8)	9/HQ
2021/Zhadina	United States (Zhadina et al., 2021)	DS	Bone	NA	49	25/49	R201H (16) R201C (9)	NA	NA	10/HQ
2021/Hagelstein	Netherlands (Hagelstein-Rotman et al., 2021)	NGS	Bone	NA	47	27/47	NA	NA	NA	9/HQ
2019/Zhang	China (Author Anynomous, 2019)	DS	Bone	NA	49	22/49	NA	NA	PFD (49)	5/MQ
2019/Romanet	France (Romanet et al., 2019)	ddPCR	Blood	NA	9	7/9	R201H (4) R201C (3)	NA	MAS (9)	9/HQ
2019/Majoor	Netherlands (Majoor et al., 2019)	NGS + DS	Myxomas	CGN + EGN (1) EGN(5)	6	5/6	R201H (4) R201C (1)	NA	PFD (5) MAS(1)	8/HQ
2019/Elli	Italy (Elli et al., 2019)	ddPCR	Bone	NA	12	8/12	NA	NA	MAS (12)	6/MQ
2017/Bas	Netherland (Majoor et al., 2018)	NGS/DS	Brest cancer, Bone	CGN + EGN(6) EGN (4)	9	4/9	R201H (3) R201C (4)	NA	PFD (6) MAS (9)	8/HQ
2017/Zuo	China (Zuo and Zhang, 2017)	NGS	Blood	CGN (3) EGN (3)	6	2/6	R201H (1) R201C(1)	NA	PFD (4) MFD (1) MAS (1)	9/HQ
2017/Shin	Korea (Shin et al., 2017)	PAP	Bone	CGN (34), EGN (53)	87	28/87	R201H (14) R201C (14)	NA	MFD (77) PFD(10)	10/HQ
2016/Cho	Korea (Cho et al., 2016)	MEMO	Blood	CGN (3) CGN + EGN (5)	8	3/8	R201H (2) R201C (1)	NA	MAS(8)	8/HQ
2016/Jour	United States (Jour et al., 2016)	DS	Bone	NA	52	26/52	R201H (17) R201C (8) R201S (1)	lg-OSA (12)	NA	NA
2016/Qin	China (Xue-yan et al., 2016)	PAP	Blood	NA	14	11/14	NA	NA	MAS(14)	9/HQ
2014/Walther	Germany (Walther et al., 2014)	DS	Bone	EGN (8)	8	5/8	R201H (3) R201C(2)	NA	NA	7/MQ
2014/Gaujoux	France (Gaujoux et al., 2014)	DS	DS	NA	6	2/6	R201C(2)	NA	MAS (6)	6/MQ
2013/Shi	China (Shi et al., 2013)	DS	Bone	CGN (25); CGN + EGN (5)	30	27/30	R201H (19) R201C (8)	OF (21) BFOL (1)	MAS (4) PFD (17) MFD (9)	NA
2013/Flore	France (Tabareau-Delalande et al., 2013)	DS	Bone	GN (10) EGN (34) NA (7)	51	23/51	R201H (12) R201C (11)	OF (21), OFD (3), OSD (1), BFOL (1) lg-OSA(14)	PFD (2) MFD (49)	NA
2013/Narumi	Japan (Narumi et al., 2013)	NGS	Blood	NA	16	10/16	R201H (6) R201C (4)	NA	MAS: 16	8.HQ
2012/Lee	Korea (Lee et al., 2012)	DS	Bone	GN (10) EGN (38)	48	28/48	R201C (25) R201H(3)	NA	MFD:40; PFD:8	8/HQ
2011/Mariot	United States (Mariot et al., 2011)	DS	Bone	NA	8	5/8	R201C(4) R201H (1)	NA	MAS: 8	8/HQ
2011/Liang	United States (Liang et al., 2011)	PAP	Bone	NA	24	23/24	R201C (4) R201H(19)	OF (10), OFD(6), PD (7), OSG (1), BFOL(3)	NA	10/HQ
2003/Corsi	Italy (Corsi et al., 2003)	DS	Bone	NA	13	13/13	R201C(10) R201H(3)	NA	MAS (11) PFD (2)	7/MQ
2008/Sergei	Italy (Kuznetsov et al., 2008)	DS	Bone	CGN (5) EGN (10)	15	15/15	R201H(3) R201C(12)	NA	NA	6/MQ
2009/Tang	China (Juan et al., 2009)	DS	Bone	12 EGN	12	2/12	R201C(1) R201H(1)	lg-OSA (10)	NA	NA
2007/Idowu	United Kingdom (Idowu et al., 2007)	DS	Bone	EGN (67); GN (5)	72	56/72	Q227L(3), R201H(32), R201C(21)	lg-OSA (3), OFD (10)	MAS (5), PFD (5), MFD(62)	NA
2007/Michienzi	United States (Michienzi et al., 2007)	DS	Bone	NA	17	14/17	R201C(12) R201H(2)	Normal Donor (12)	MAS (15) PDF (1) MFD(1)	9/HQ
2007/Toyosawa	Japan (Toyosawa et al., 2007)	CS	Bone	CGN (5) EGN (4)	9	9/9	R201H(4) R201C(5)	OF(5)	MFD (8) PFD(1)	NA
2006/Kobayashi	Japan (Kobayashi et al., 2006)	CS	Bone	NA	16	16/16	R201C(7) R201H(9)	NA	MFD:13 PFD:3	6/MQ
2006/Kalfa	France (Kalfa et al., 2006)	DS	Bone	Bone	15	12/15	NA	NA	MAS(15)	10/HQ
2001/Pollandt	Germany (Pollandt et al., 2001)	DS	Bone	9 EGN	9	9/9	R201H (6) R201C(3)	lg-OSA (5)	MFD (9)	NA
2000/Bianco	United States (Bianco et al., 2000)	DS	Bone	CGN (6) EGN (2)	8	8/8	R201H (4) R201C (4)	NA	PFD (5) MFD (3)	8/HQ
2000/Sakamoto	Japan (Sakamoto et al., 2000)	DS	Bone	NA	7	7/7	R201H (3) R201C(4)	OFD (7) Normal Donor (1)	MFD (6) PFD(1)	NA
1999/Stanton	United States (Stanton et al., 1999)	DS	Bone	NA	11	10/11	R201H (5) R201C(5)	NA	MAS (6) PFD (2) MFD(3)	6/MQ
1997/Candeliere	Canada (Candeliere et al., 1997)	DS	Bone	9 EGN	9	8/9	R201C (3) R201H (4) R201S (1)	NA	PFD (6) MFD (1) PAFD(2)	7/MQ

Notes/Abbreations: NA, not available; No., number; FD, fibrous dysplasia. Location: CGN, Cranial-gnathic; EGN, extra-gnathic. AHRQ, Score; AHRQ, agency for healthcare research and quality; HQ, high quality; MQ, moderate quality. FD, type; MFD, monostotic fibrous dysplasia; PFD, polyostotic fibrous dysplasia; MAS, McCune–Albright syndrome. Detection method: MEMO, Mutant enrichment with 3′-modified oligonucleotides; DS, direct sequencing; NGS, next-generation sequencing; PAP, pyrophosphorolysis activated polymerization; CS, clone sequencing; ddPCR, digital droplet polymerase chain reaction. Other diseases: OF, ossifying fibroma; OFD, osteofibrous dysplasia; OSD, osseous dysplasia; lg-OSA, low-grade osteosarcoma; OSG, osteogenesis imperfecta; PD, paget disease; BFOL, fibro-osseous lesion; DSOM, chronic diffuse sclerosing osteomyelitis.

### 3.2 Quantitative synthesis

#### 3.2.1 Prevalence of *GNAS* mutations in FD

**FIGURE 3:**
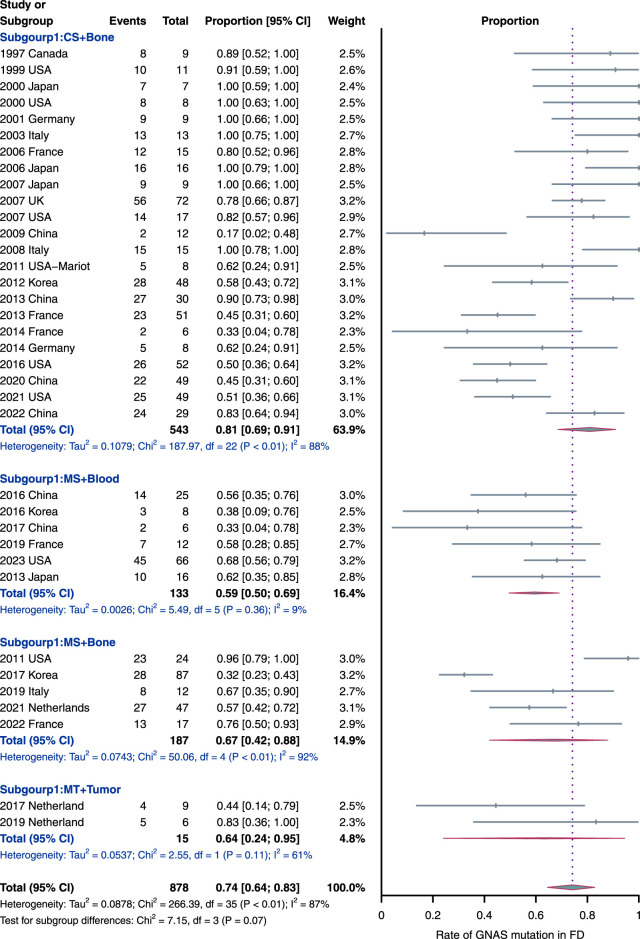
Rate of *GNAS* mutation detection in FD samples. The subgroups were as follow: MS, modified sequencing methods, including pyrophosphorolysis activated polymerization, digital droplet polymerase chain reaction, mutant enrichment with 3′-modified oligonucleotides and next-generation sequencing methods; CS, conventional sequencing methods, including direct or clone sequencing methods; and MT, multiple sequencing methods used in each study.

The *GNAS* mutation data for FD patients were available for 36 studies (*n* = 878 patients). The overall pooled prevalence of *GNAS* mutations in FD patients based on the random effects model was 74% (95% CI = 64%–83%, Figure 3), with a high heterogeneity (*I* ^2^ = 87%, *p* < 0.01). The observed heterogeneity can be attributed to two factors, namely, diverse sample types for DNA acquisition and distinct sequencing methods. To address this, a subgroup analysis was conducted based on the sample types and sequencing methods. This analysis revealed four subgroups: conventional sequencing with bone tissues (CS + Bone), modified sequencing with bone tissue (MS + Bone), modified sequencing with blood (MS + Blood), and multiple sequencing methods with tumor tissue (MT + Tumor).

Notably, the “CS + Bone” subgroup emerged as the largest, comprising 543 samples from 23 studies, suggesting its current prevalence. This preference may be attributed to its straightforward process and cost-effectiveness. Simultaneously, the “CS + Bone” subgroup exhibited the highest prevalence of *GNAS* mutations (81%, 95% CI = 69%–91%) compared to the other subgroups. Additionally, it is worth highlighting that the “MS + Blood” subgroup (*n* = 133; six studies) displayed the lowest prevalence of *GNAS* mutations (59%, 95% CI = 50% to 69%) with a very low heterogeneity (*I* ^2^ = 9%, *p* = 0.36), diverging significantly from the overall pooled outcomes mentioned earlier. The prevalence of *GNAS* mutations in ‘MS + Bone’ and ‘MT + Tumor’ subgroups was 67% (95% CI = 42%–88%; n = 187) and 64% (95% CI = 24%–95%; *n* = 15; two studies), respectively. In addition, we also performed an unpaired *t*-test to compare the detection rate between “CS + Bone” and “MS + Bone,” and there is no statistically significant difference (*p*-value > 0.05, Supplementary Material).

#### 3.2.2 Diagnostic accuracy of *GNAS* mutations for FD

In total, 10 of 35 included studies containing differentiated cases of FD were included for this diagnostic accuracy evaluation analysis (in the ninth column of Table 1). In addition, normal donors and patients in the control group were not included in this meta-analysis, as this could have exaggerated the accuracy of the diagnoses. All included studies had a negative correlation coefficient of the threshold effect (*r* = 0.52, *p* = 0.11).

**FIGURE 4:**
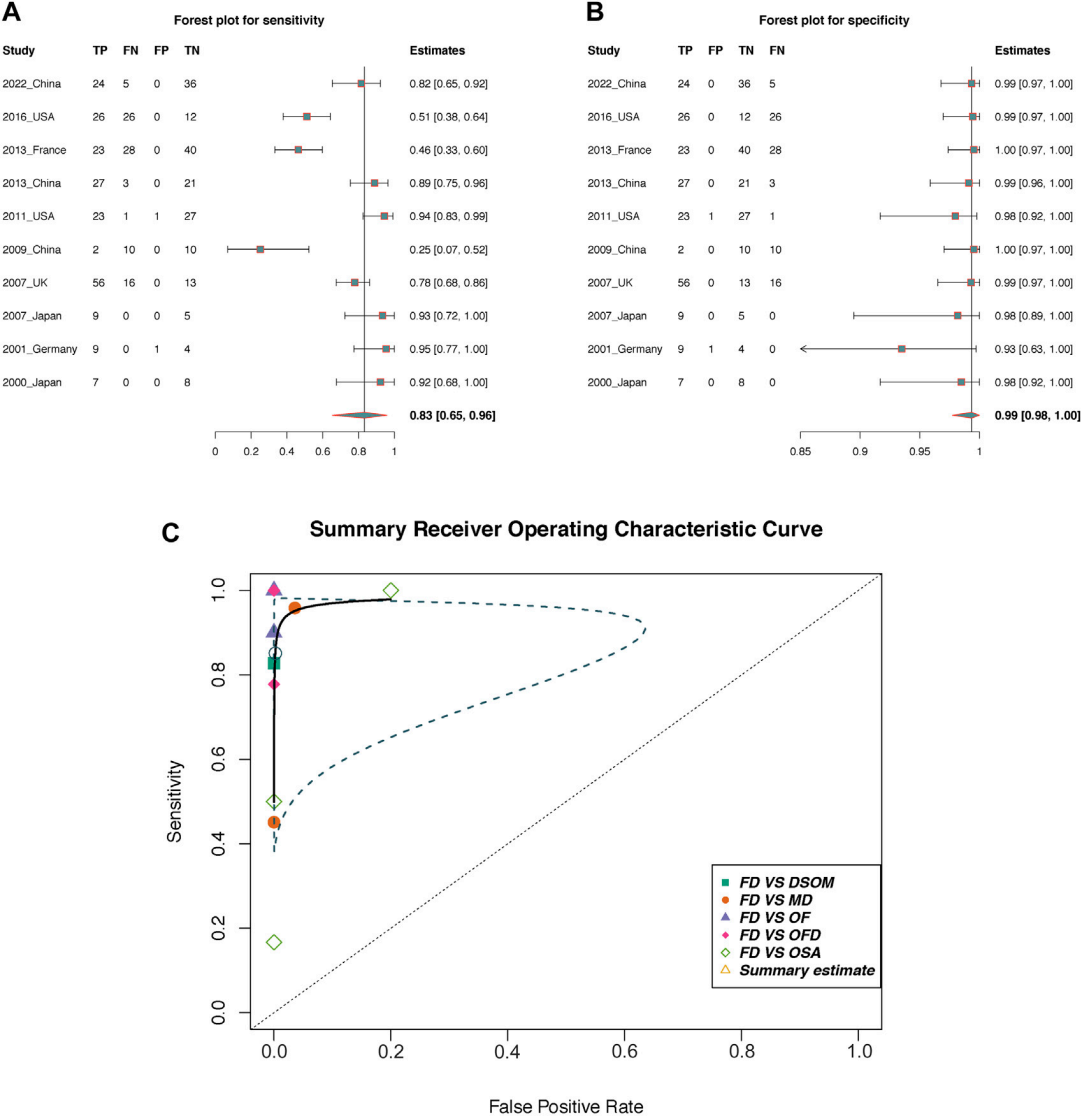
The diagnostic accuracy of *GNAS* mutation in fibrous dysplasia. **(A,B)** Forest plots of sensitivity and specificity; **(C)** Summary receiver operating curve (SROC). TP, True positive; FP, False positive; TN, True negative; FN, False negative; OF, Ossifying fibroma; OFD, osteofibrous dysplasia; OSA, low-grade osteosarcoma; DSOM, chronic diffuse sclerosing osteomyelitis; MD, multiple diseases; Blue dotted line area is the confidence interval.

**FIGURE 5:**
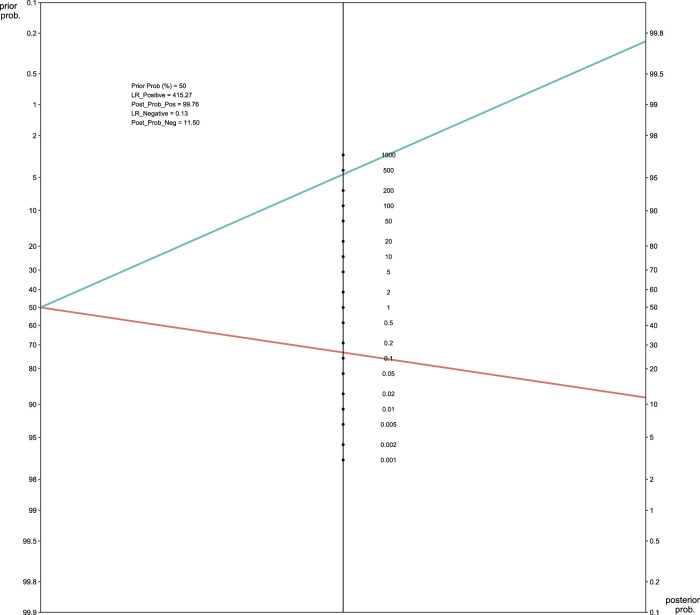
Fagan’s normogram of *GNAS* mutation in FD. LR, likelihood ratio.

The pooled sensitivity and the 95% confidence interval for *GNAS* mutations for the FD were 0.83 (95% = 0.65 to 0.96, Figure 4A) while the pooled specificity was 0.99 (95% = 0.98 to 1, Figure 4B). The positive likelihood ratio (LR+) was 415.27 (95% CI, 13.82 to 1,555.30, Supplement Figure 1A), the negative likelihood ratio (LR-) was 0.13 (95% CI, 0.07 to 0.19, Supplement Figure 1B), and the diagnostic odds ratio by log was 7.23 (95% = 4.33 to 9.08, Supplement Figure 1C). The area under the SROC curve was 98.38% (Figure 4C), which indicates excellent for a diagnostic test (Mandrekar, 2010). Figure 5 displayed the Fagan’s Normogram, showing that the post-test probability of LR+ is 99.76%, whereas the post-test probability of LR-is 11.5%.

#### 3.2.3 Genotype-phenotype correlations in FD

**FIGURE 6:**
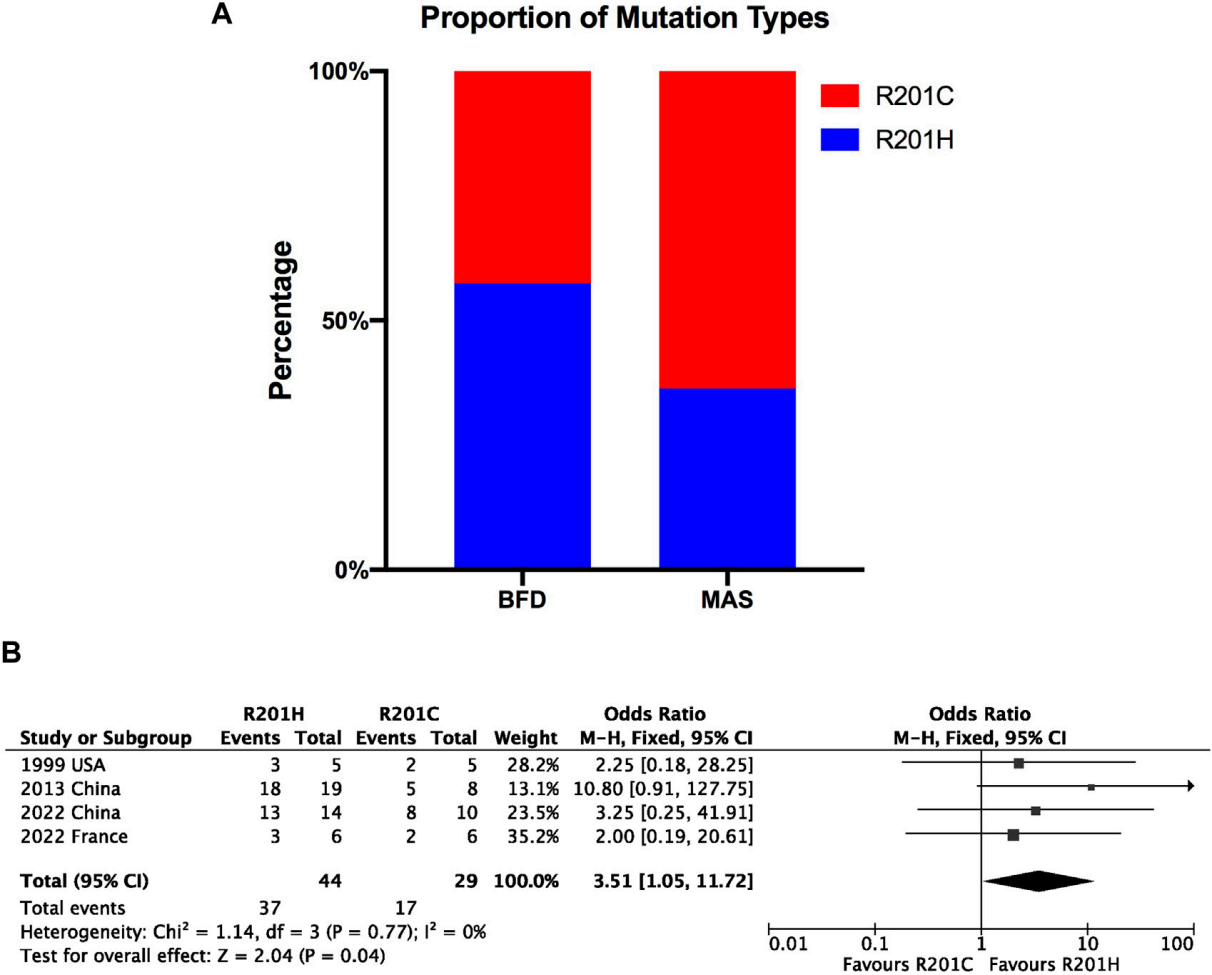
Bar plot for proportion of mutation types (R201H and R201C) in BFD and MAS **(A)** and forest plot for genotype-phenotype correlation in FD **(B)**.

To investigate the correlation between the genotypes and phenotypes of FD/MAS, we divided FD into two categories: FD with lesions only in bone sites (BFD, including MFD and PFD) and MAS. Therefore, we investigated whether R201H mutations were more frequent on BFD than were R201C mutations. The occurrence and fraction of R201H and R201C in BFD and MAS are shown in Table 2 and Figure 6A, and the Fisher’s exact test result revealed a significant association of different mutation types with different FD types (*p* < 0.05, Table 2), and R201H mainly occurred in BFD while R201C mainly occurred in MAS (Figure 6A).

**TABLE 2:** Correlation between the mutation types and FD types.

	R201H	R201C	*p*-value (Fisher’s exact Test)
BFD	96	71	0.0349
MAS	12	21	

Notes/Abbreations: BFD, FD, with lesions only in bone sites; MAS, McCune–Albright syndrome.

We also included four studies that simultaneously recorded the specific mutation types in BFD and MAS for meta-analysis. The pooled results also showed a significant association of different mutation types with different FD types (OR = 3.51; 95% CI = 1.05 to 11.72; *p* = 0.04) with no heterogeneity (Figure 6B), but after the sensitivity analysis, the data were not stable, especially after excluding the 2013 China study (Shi et al., 2013), after which the association was not significant (OR = 2.42; 95% CI = 0.58 to 10; *p* = 0.96); this indicates that more relevant evidence is still needed.

## 4 Discussion

Mutation detection methods can be a reliable assistant tool for pathologic diagnosis when confusing microscopic features are observed (Delaney et al., 2009), and currently FD has been found to uniquely contain *GNAS* mutations. The *GNAS* mutations were first identified in patients with McCune-Albright syndrome in 1991 (DiCaprio and Enneking, 2005). Since then, more studies have continued to update the incidence data of *GNAS* mutations in FD. In this study, we found that the pooled prevalence of *GNAS* mutations in FD patients was 74% (95% CI, 64%–83%), which was obtained from 34 articles published from 1997 to 2022. A mutation with such a high incidence can provide us with an understanding of FD and plays a pivotal role in two aspects: understanding the disease mechanism and enabling diagnosis. Regarding the mechanism, the current view is that *GNAS* mutations can cause a disruption in GTPase activity and constitutive Gs-α activation (Robinson et al., 2016), which leads to the overproduction of cAMP that causes hyperproliferation and incomplete differentiation of marrow stromal cells to abnormal osteoblasts in FD and inhibits osteoblasts-specific genes as well as stimulating cytokines that promote bone resorption by osteoclasts (Riddle and Bui, 2013).

While the overall pooled prevalence appears elevated, it is important to observe, as indicated by the forest plot, that the mutation rates in certain cohorts fall below the 50% threshold. For negative results, it cannot be concluded that the patient does not have a *GNAS* mutation status, as FD is a mosaic disease, and the location of cells with DNA mutations is not uniform. In certain instances, the presence of undetectable *GNAS* mutations in FD cases might find an explanation in the mosaic nature of FD. This mosaic pattern entails lower proportions of mutated cells when juxtaposed with their non-mutated counterparts. Alternatively, such occurrences could be attributed to the emergence of novel mutations within the context of fibrous dysplasia. This diversity in genetic landscapes could contribute to instances where *GNAS* mutations remain elusive in the diagnostic process. Therefore, the detection rate of *GNAS* is somewhat unstable because we do not know the proportion of tissue obtained that is occupied by cells rich in *GNAS* mutations. In addition, an alternative perspective posits that sequencing methods can impact the detection rate of *GNAS*, including the conventional direct sequencing approach. This method necessitates DNA of high quality and quantity, coupled with a mutant threshold of approximately 20% within the total population (Liang et al., 2011). However, controversy surrounds this viewpoint. Presently, the prevailing belief is that the variable influencing sequencing technology’s impact on detection rates lies in the diverse sources of DNA. Imanaka et al. (Imanaka et al., 2007) suggested subjecting DNA from blood to more advanced sequencing methods, such as PAP, NGS, and ddPCR. This recommendation arises from the inability of direct sequencing to detect mutations in peripheral blood leukocytes adequately (de Sanctis et al., 2017). Therefore, in comparison to bone tissue, employing a modified sequencing method in blood significantly improves the detection rate of *GNAS* mutations. Interestingly, in this study, those literature extracting DNA from peripheral blood all employed a modified sequencing method as opposed to DS, aligning with the aforementioned theoretical viewpoint. Furthermore, within the “DS + Bone” subgroup, serveral cohorts still exhibited a detection rate of less than 50%, highlighting mosaicism of FD as a significant factor contributing to negative results.

In the included literature, the utilization of modified sequencing technology for FD has progressively emerged since 2011. However, based on the findings of this study, the inherent mosaicism of FD and the cost-effective achievement of 80% accuracy by conventional direct sequencing, the authors contend that conventional direct sequencing remains the optimal method for molecular detection of FD when the specimen is bone. For the tissue selection, the bone affected by FD would be the most appropriate tissue source for detecting DNA mutations in FD patients (Candeliere et al., 1997). Given that it serves as the primary site of the ailment, the concentration of mutations therein will be at its peak. In addition, several studies have attempted to ascertain GNAS mutations by analyzing skin or thyroid tissue samples (Xing et al., 2022). However, given the paucity of relevant literature and data, these analyses were not incorporated into the present study. In this study, the detection rate of *GNAS* mutations in bone tissue was higher than that in blood tissue, which indicated that bone is a more effective tissue for *GNAS* mutation testing. In conclusion, the conventional direct sequencing method, when combined with bone tissue, represents the optimal choice.

Regarding diagnosis, a differential diagnosis between FD and many bone lesions, whose specific diseases have been described in the introduction, is needed. Taking DSMO and OF as examples, the overlapping clinicopathological features that appear in some of special cases could lead to difficulties in obtaining a diagnosis, and the therapies and prognoses of these two diseases are extremely different (Shi et al., 2013; Xue et al., 2022). Therefore, it is essential to accurately distinguish FD from other bone lesions. However, the specific diagnostic accuracy of the *GNAS* mutation detection method is still unclear. In this study, the pooled sensitivity was 83%, the specificity was almost 100%, and the specificity was more consistent than the sensitivity, which means that *GNAS* mutations are probably a unique characteristic in FD, as other bone lesions do not possess *GNAS* mutations. Furthermore, low-grade central osteosarcoma (lgcOSA) is one of the most important differential diagnoses of FD (Walther et al., 2014), this study showed that only one lgcOSA case (1/15) had a *GNAS* mutation, which also indicates the ideal unique specificity of *GNAS* mutations in FD. The SROC in this study showed a great value of 98.5%, which indicates that *GNAS* mutation detection is a reliable diagnostic method. However, from the results' perspective, the sensitivity is less stable than the specificity, aligning with the previously discussed the overall pooled rate. In essence, when the detection outcome is wild type, additional discrimination measures are necessary due to the mosaicism of FD. In addition, the value of LR+ > 10 or LR− < 1 indicates the more likely or less likely the disease occurred, respectively. This finding concluded that *GNAS* mutation has an increase and slight decrease in the likelihood of detecting FD, with the value of LR + being 415.27 and LR− of 0.13, correspondingly.

*GNAS* mutations in FD mainly include R201H (arginine replaced by histidine) and R201C (arginine replaced by cysteine). In this study, there was a significant association between genotypes and different phenotypes of FD/MAS. More specifically, R201H mutations mainly occur in FD confined to the bone site, and R201C mutations mainly occur in MAS. However, this finding needs to be updated in the future, as the results were unstable after sensitivity analysis. The R201H variant may confer a selective advantage by enhancing the proliferative or survival capacity of affected cells *in vivo* (Landis et al., 1989). Theoretically, it is difficult to facilitate a preference for developing one mutation type over the other via a biochemical mechanism because R201C and R201H variants in *GNAS* consistently occur in the same codons (CGT-TGT and CGT-CAT, respectively) (Riminucci et al., 2006). Therefore, more evidence is warranted to further explore the genotype-phenotype correlation in FD.

Several reasons can explain the heterogeneity observed in this study. Different sample sizes could lead to instability in the prevalence results, especially when the sample sizes are small. In this study, the prevalence of *GNAS* mutations in subgroups with large samples (65%) was lower than that in the subgroups with moderate and small sample sizes (85% and 86%, respectively), but none of them deviated significantly from the scope of the overall result. Additionally, different mutation detection methods and sources of DNA acquisition could also lead to heterogeneity. For the detection methods, the direct sequence technique was mostly applied in this study, but it can be matched with different PCR technologies, such as conventional PCR, nested PCR and protein nucleic acid-based PCR. However, there is still no comprehensive study to explore the specific differences in *GNAS* mutation detection rate between PCR with DS and other detection methods, including ddPCR, PAP and NGS, which could be an interesting factor to consider in selecting a detection method for *GNAS* mutations in the future.

To our knowledge, this study is the first to investigate the prevalence, diagnostic accuracy and the genotype-phenotype correlation of *GNAS* mutations in FD patients. However, there are a few limitations that inevitably should be concerned. First, the sample size of the included studies was inconsistent. Since FD is a rare disease, some small sample studies were included, and the proportion of *GNAS* mutations in several of these small sample studies was up to 100%, so larger studies are needed, preferably studies with sample sizes greater than 30, to update the *GNAS* mutation rate. Second, the included studies failed to evaluate the impact of different sample sources or sequencing methods on the detection rate for *GNAS* mutations, as barely studies evaluated the detection rate in different sequence methods or sample sources by comparing with control groups. This issue will be the focus of research on the efficacy of *GNAS* detection. Therefore, this article can only explore their differences vertically and cannot compare them horizontally. Third, the included literature of exploring the genotype-phenotype correlation in different FD types, especially MAS, is limited, as many included studies did not report the occurrence of specific *GNAS* mutations for corresponding FD/MAS, so more studies containing classification records are warranted. Finally, the included studies were all observational, which can have associated biases, such as selection bias. Finally, this study was unable to discuss the incidence of other rare mutation types, such as Q227 (Idowu et al., 2007), which were not included in this article for analysis due to insufficient records.

## 5 Conclusion

In conclusion, this study showed that *GNAS* mutations have a very high pooled prevalence of 74% in FD, which indicates that these mutations are responsible for a larger proportion of FD patients, and pooled diagnostic accuracy analysis showed that *GNAS* mutation detection is a reliable and efficient method for differential diagnosis from other bone lesions that can help doctors achieve early and accurate detection of FD in unclear cases. However, the detection rate of *GNAS* mutations exhibits instability, mirroring the mosaicism of FD. Consequently, in cases where a negative result is obtained, alternative methods must be employed to pursue further identification. Additionally, we preliminarily found a significant association between mutation variants in FD types, which is essential for the future development of targeted therapies.

## Data Availability

The original contributions presented in the study are included in the article/Supplementary Material, further inquiries can be directed to the corresponding authors.
