# Supplementary figures and images for: The prevalence, diagnostic accuracy and genotype-phenotype correlation of GNAS mutations in fibrous dysplasia: a meta-analysis
Source: Front Genet. 2024 Jul 29;15:1377716. doi: 10.3389/fgene.2024.1377716 (PMC11317392; doi:10.3389/fgene.2024.1377716)

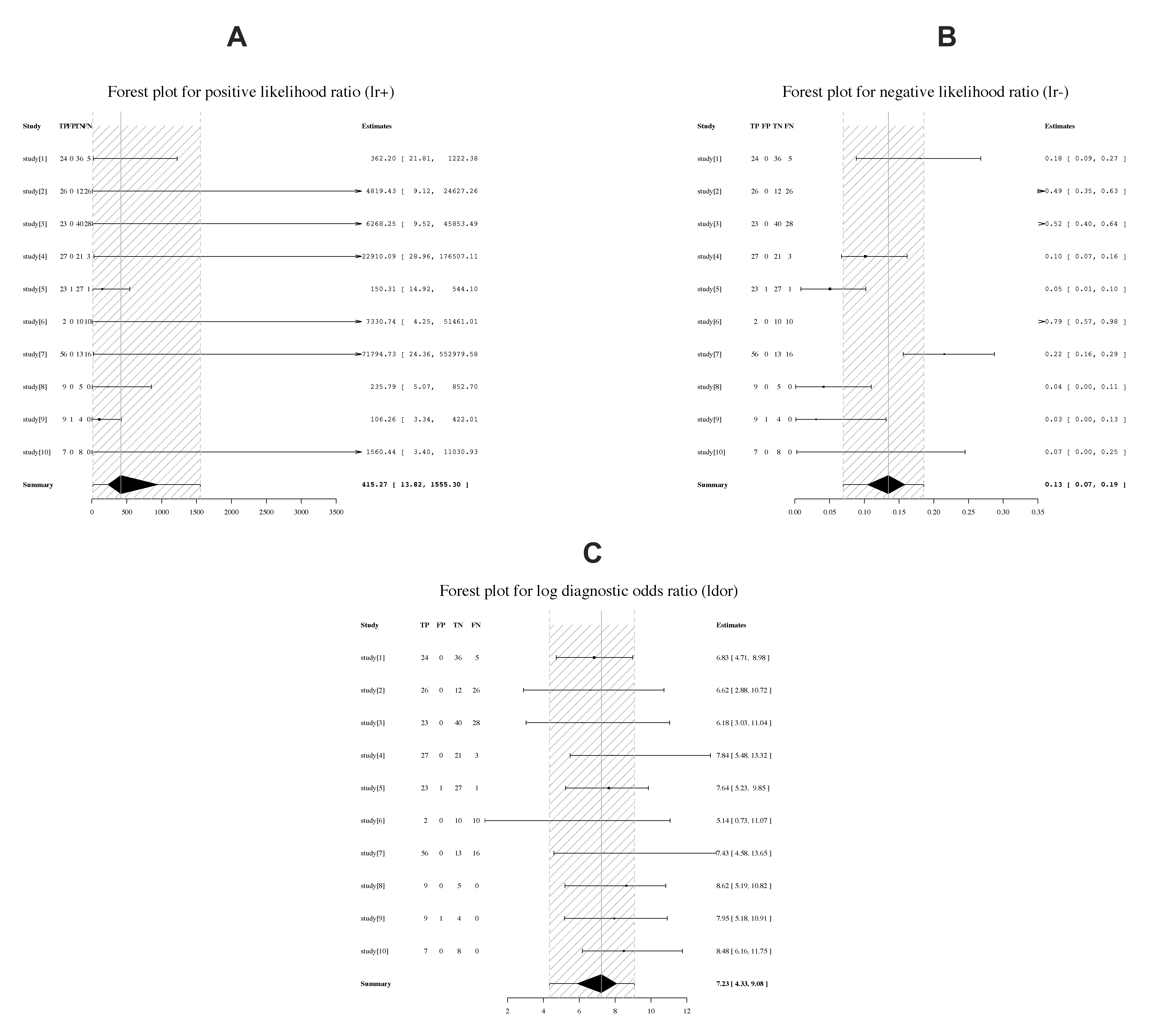

Supplement: Supplementary file 1 [file Image1.JPEG]
